# Identification of Clinical Phenotypes Among People with HIV Using Electronic Health Record Data

**DOI:** 10.1007/s10461-025-04893-7

**Published:** 2025-10-06

**Authors:** Anoop Mayampurath, Sheriff Isakka, Joseph A. Mason, Samuel Nycklemoe, Eleanor E. Friedman, Jessica P. Ridgway

**Affiliations:** 1 Department of Biostatistics & Medical Informatics, University of Wisconsin-Madison, Madison, WI USA; 2 Department of Surgery, University of Wisconsin-Madison, Madison, WI USA; 3 Department of Medicine, University of Chicago, Chicago, IL USA

**Keywords:** Clinical phenotypes, Retention in care, HIV care continuum, Viral suppression

## Abstract

Loss to follow-up (LTFU) from HIV care affects nearly half of people with HIV (PWH). This study aimed to identify clinical phenotypes of PWH using electronic health record (EHR) data and assess their association with LTFU. We analyzed 4,316 visits from 849 adults receiving care at an urban Ryan White Clinic (2017–2020), extracting demographic, social history, laboratory, diagnosis, and clinical note features. Latent Class Analysis identified six distinct patient subgroups, and logistic regression assessed their association with LTFU and high viral load within one year of a clinic visit. Analysis of clinical phenotypes revealed the following characteristics: Class 1: higher rates of substance use and cardiovascular disease and mentions of “heterosexual” in notes; Class 2: younger men, new patients, highest rate of LTFU (OR 1.57, 95% CI 1.20–2.05) and elevated viral load (OR 1.74, 95% CI 1.42–2.13); Class 3: primarily women, with frequent mentions of “pregnancy” in notes; Class 4: more likely to be white or Hispanic, fewer mentions of “substance use disorder” in notes, increased LTFU risk (OR 1.39, 95% CI 1.02–1.89) and decreased risk of unsuppressed HIV viral load (OR 0.65, 95% CI 0.50–0.84); Class 5: more sexually active, with mentions of “good adherence” in notes; Class 6: older men, with fewer mentions of “mental illness” or “STI” in notes. In conclusion, we identified six clinically relevant phenotypes in an urban HIV clinic, two of which were associated with LTFU. Our findings could inform strategies to tailor interventions to improve retention in HIV care for PWH.

**Supplementary Information:**

The online version contains supplementary material available at 10.1007/s10461-025-04893-7.

## Introduction

People with HIV (PWH) who are engaged in consistent medical care are more likely to receive antiretroviral therapy, have decreased mortality, and are less likely to transmit HIV than those not retained in care [1, 2]. Despite these benefits, only half of PWH in the United States are retained in care. Therefore, improving care retention among PWH is critical for addressing the HIV epidemic in the U.S.

Basic interventions to improve retention in care, such as appointment reminders and clinic-based retention messages, have met limited success. These interventions yield modest improvement in visit attendance [3,4]. Researchers have identified successful interventions, such as intensive case management, peer navigation, and multi-faceted outreach programs [5–10]. Because these interventions are resource-intensive, we and others have developed algorithms to identify patients most vulnerable to loss to follow-up (LTFU) who would most benefit from retention-focused resources before LTFU occurs [11–15].

While these studies are promising, successfully translating these models and accompanying interventions into positive outcomes for PWH hinges on equivalent model performance across all patients. However, PWH are a markedly heterogeneous population as HIV impacts people differently based on a spectrum of co-occurring conditions (such as social drivers of health, substance use, mental health, stigma, other diseases, etc.). Studies have demonstrated that certain interventions prove less effective among PWH experiencing poverty, housing instability, and among Black and Hispanic populations [19–21]. Therefore, identification and intervention for PWH at risk for not being retained in care is not a ‘one-size-fits-all’ approach but a more tailored approach for sub-groups of patients that differ drastically in the factors that are predictive of LTFU.

While studies have focused on identifying subgroups of PWH based on molecular-level analysis of HIV-1 [16–18], a data-driven approach to identifying clinical phenotypes of PWH using electronic health record (EHR) data has not been undertaken so far. Several studies have highlighted how the use of phenotyping methods based on unsupervised clustering could be used to detect clinical phenotypes in diseases such as substance use disorders or stroke [19–21]. Similar approaches could be used to detect clinical phenotypes of PWH that differ in terms of their characteristics, adverse health outcomes, etc., thereby holding the potential for increasing the practice of personalized care in PWH.

The aim of this study is to identify clinical phenotypes of PWH using EHR structured (e.g., labs, diagnoses) and unstructured (e.g., clinical notes) data and understand differences in terms of clinically meaningful traits and risk for LTFU. We hypothesize that unsupervised learning algorithms such as latent class analysis (LCA) can be used to detect PWH clinical phenotypes that differ in retention rates and HIV viral load.

## Methods

### Study Population and Data Sources

We conducted a retrospective analysis of adult (age ≥ 18 years) PWH who attended at least one medical appointment with an HIV provider at an urban Ryan White HIV care clinic between January 1, 2017, and December 31, 2020. The clinic is located on the south side of Chicago, which has high HIV prevalence [22] and is in Cook County, an Ending the HIV Epidemic (EHE) jurisdiction. We extracted demographic characteristics, social history, laboratory results, and ICD-10 diagnosis codes for each clinic visit. ICD-10 diagnosis codes were collected from the encounter-level diagnoses and the problem list during the study period. We also extracted clinical notes from the visit written by physicians, advanced practice providers, nurses, social workers, and case managers. The study was approved by our institution’s Institutional Review Board with a granted waiver of consent.

### Variables used for Phenotyping

For the phenotyping, we considered demographic characteristics, visit-related diagnosis codes, social history, laboratory results, history of prior LTFU, and text features extracted from clinical notes. All included features have been established through prior work as being important to PWH care retention [11–13, 23]. Age was binned into five categories (≤ 25, 26–35, 36–45, 46–55, and > 55 years) based on clinical relevance and sample size. We utilized 27 key diagnosis variables, encompassing a wide range of conditions, including substance use disorders, mental health issues, chronic diseases, and sexually transmitted infections. The complete list of EHR-related variables is given in Supplementary Table  1.

Clinical notes were searched using natural language processing techniques to identify mentions of specific word tokens related to predefined clinical topics. These clinical topics were highlighted in our prior work as important for predicting retention [23], and the approach was replicated in this study. The complete list of clinical topics, associated tokens, and exclusion tokens is given in Supplementary Table 2. Each clinical topic was marked as present for a visit if any associated token was found in the notes and did not have mentions of exclusion terms (e.g., “no,” “neg,” etc.) in the surrounding ± 3 words. A clinical topic was marked as absent for a visit if none of the associated tokens were found or token mentions were excluded based on mentions of exclusion terms. Only exact matching was used to search for terms. Extending the exclusion to ± 5 words did not change results.

### Phenotyping of PWH

We employed Latent Class Analysis (LCA), a probabilistic modeling technique that estimates the likelihood of individuals belonging to mutually exclusive latent classes. This approach allowed us to identify subgroups within the overall heterogeneous cohort of PWH using our variables while providing both subgroup assignments and probabilities of subgroup membership for each individual. PWH assignment to a single latent class was based on using the maximum probability of class membership. We tested models with varying numbers of latent classes, ranging from one to ten. All variables listed in Supplementary Tables 1 and 2 were used for the LCA. To ensure robustness in determining an unbiased optimal number of subgroups, we employed multiple model fit statistics, including the Bayesian Information Criterion (BIC), Akaike Information Criterion (AIC), Sample-size Adjusted BIC (SABIC), and Consistent AIC (CAIC), to determine the best model. No other metrics were used for model selection. We did not test the LCA using a reduced number of variables, as these are likely to bias our results.

### Clinical Outcomes

Our primary outcome of interest was LTFU. We defined LTFU as more than 365 days elapsing between consecutive HIV clinic appointments that were attended [24]. Our choice of primary outcome was based on prior studies [11]. We also considered a secondary outcome of high viral load, defined as an HIV viral load being ≥ 200 copies per ml from samples drawn during visits in the 365 days following an HIV clinic appointment. Both outcomes were calculated at the visit level. We compared the rates of primary and secondary outcomes across detected clinical phenotypes. Finally, we considered an alternative definition of LTFU, the National HIV/AIDS Strategy (NHAS) outcome, in which a patient was considered retained in care if the patient attended at least 2 HIV care visits greater than 90 days apart within a 365-day period [25, 26].

### Statistical Analysis

We used chi-squared and Kruskal–Wallis statistical tests to compare categorical and numeric variables, respectively, between patients who were LTFU and those who were ever retained throughout the study period. Statistical comparison of variables between the clinical phenotypes was also performed using chi-squared tests. We also created single-variable logistic regression models to determine the association between class membership and the likelihood of experiencing each outcome. All analyses were conducted using Python version 3.12.2, with two-sided P < 0.001 values denoting statistical significance.

## Results

### Study Cohort

The study cohort consisted of 849 individuals with 4,316 visits with an HIV care provider between 2017 and 2020, of which 286 (33.7%) were consistently retained in care using our LTFU outcome. Patients retained at any point in the study period were similar to LTFU patients in age, sex, race, and ethnicity (see Table 1). However, patients who were LTFU had a lower median number of clinic visits (3 vs. 8, P < 0.001) and a higher proportion of patients with an elevated most recent viral load (79% vs. 61%, P = 0.009) compared to patients who were retained throughout the study period.

**Table 1:** Comparison of characteristics between patients who were lost to follow-up at any point during the study period compared to those fully retained

	Patients who were lost to follow-up during study period (n = 563)	Patients who were fully retained in study period (n = 286)	P-value
Age, years, median (IQR)	49 (29–53)	44 (29–56)	0.181
Sex*, n (%)			
Male	393 (69.8)	188 (65.7)	0.259
Female	170 (30.2)	98 (34.3)	
Race, n (%)			
White	63 (11.2)	33 (11.5)	0.201
Black	482 (85.6)	245 (85.7)	
Asian	5 (0.9)	0 (0)	
American Indian or Alaska Native	1 (0.2)	0 (0)	
Native Hawaiian or Other Pacific Islander	1 (0.2)	0 (0)	
More than one race	10 (1.8)	4 (1.4)	
Declined to respond	1 (0.2)	4 (1.4)	
Hispanic, n (%)	20 (3.6)	12 (4.2)	0.784
Number of clinic visits during study period, Median (IQR)	3.0 (2.0–7.0)	8.0 (4.0–11.0)	< 0.001
Most recent viral load ≥ 200 copies per ml, n (%)	79 (14.0)	61 (21.3)	0.009

*Based on administrative record

### Phenotype Identification

**Fig. 1:**
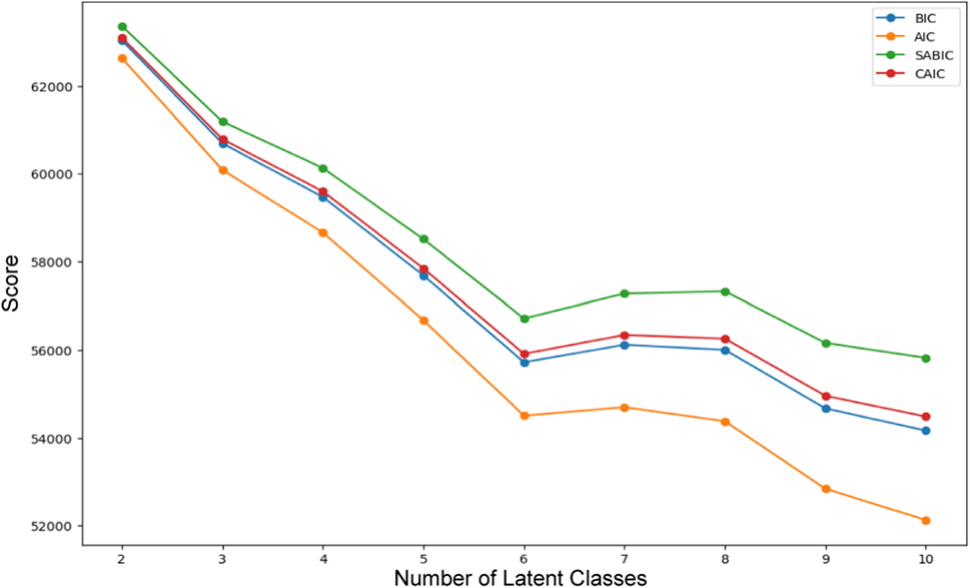
The optimal number of latent classes was selected using fit statistics, including the Bayesian information criterion (BIC), Akaike information criterion (AIC), Sample-size adjusted BIC (SABIC), and Consistent AIC (CAIC)

All fit statistics from the LCA analysis indicated that a six-latent-class model (out of models with classes ranging from one to ten) was optimal, as improvements to all fit criteria began to diminish around seven latent classes (see Fig. 1). Although model fit improved slightly when considering nine or ten latent classes, the six-class (or six-phenotype) model demonstrated meaningful differences with a better sample size distribution and improved interpretability.

Table 2 depicts the distribution of demographics, diagnoses, social history, sexual history, social history of substance use, and retention history among the six phenotypes. Table 3 shows the distribution of clinical topics among the six phenotypes. The 4316 patient visits were categorized within one of the six latent classes. Class 1 represents patients who have higher proportions of a documented social history of substance use, including using alcohol, tobacco, illicit drugs, or injection drugs. They also had a higher proportion of cardio-pulmonary diseases (cardiovascular disease, pulmonary conditions, and hypertension) as comorbidities compared to the overall cohort and other classes. Class 1 patients, representing 20% of the overall cohort, have a higher proportion of a documented record of female partners in their social history and have more mentions of the topic “heterosexual” compared to other classes.

**Table 2:** Distribution of patient characteristics in the overall cohort and among the six clinical phenotypes

	Overall (n = 4316)	Class 1 (n = 874)	Class 2 (n = 804)	Class 3 (n = 670)	Class 4 (n = 511)	Class 5 (n = 758)	Class 6 (n = 699)
Age, n (%)*							
< = 25	401 (9.3)	69 (7.9)	185 (23.0)	66 (9.9)	34 (6.7)	47 (6.2)	0 (0%)
26–35	1136 (26.3)	202 (23.1)	428 (53.2)	155 (23.1)	70 (13.7)	261 (34.4)	20 (2.9)
36–45	627 (14.5)	102 (11.7)	68 (8.5)	111 (16.6)	88 (17.2)	132 (17.4)	126 (18.0)
46–55	968 (22.4)	182 (20.8)	88 (10.9)	153 (22.8)	139 (27.2)	186 (24.5)	220 (31.5)
> 55	1184 (27.4)	319 (36.5)	35 (4.4)	185 (27.6)	180 (35.2)	132 (17.4)	333 (47.6)
Male, n (%)*	2867 (66.4)	583 (66.7)	804 (100.0)	0 (0)	465 (91.0)	316 (41.7)	699 (100.0)
Race, n (%)*							
Black	3708 (85.9)	828 (94.7)	804 (100.0)	669 (99.9)	32 (6.3)	676 (89.2)	699 (100.0)
White	481 (11.1)	34 (3.9)	0 (0)	1 (0.1)	390 (76.3)	56 (7.4)	0 (0)
Other	127 (30.0)	12 (1.4)	0 (0)	0 (0)	89 (17.4)	26 (3.4)	0 (0)
Hispanic, n (%)*	170 (3.9)	0 (0)	0 (0)	5 (0.7)	146 (28.6)	19 (2.5)	0 (0)
Diagnosis of Comorbidities, n (%)							
Cancer*	315 (7.3)	58 (6.6)	10 (1.2)	43 (6.4)	51 (10.0)	64 (8.4)	89 (12.7)
Cardiovascular disease*	408 (9.5)	145 (16.6)	28 (3.5)	27 (4.0)	48 (9.4)	72 (9.5)	88 (12.6)
Diabetes*	218 (5.1)	71 (8.1)	15 (1.9)	25 (3.7)	14 (2.7)	22 (2.9)	71 (10.2)
Pulmonary conditions*	132 (3.1)	53 (6.1)	3 (0.4)	35 (5.2)	19 (3.7)	5 (0.7)	17 (2.4)
Hypertension*	1071 (24.8)	274 (31.4)	77 (9.6)	183 (27.3)	112 (21.9)	160 (21.1)	265 (37.9)
Kidney disease*	252 (5.8)	56 (6.4)	36 (4.5)	41 (6.1)	5 (1.0)	48 (6.3)	66 (9.4)
Liver disease*	358 (8.3)	56 (6.4)	0 (0)	55 (8.2)	52 (10.2)	30 (4.0)	165 (23.6)
Pregnancy, n (%)*	139 (3.2)	10 (1.1)	0 (0)	69 (10.3)	0 (0)	60 (7.9)	0 (0)
Obesity, n (%)*	394 (9.1)	72 (8.2)	34 (4.2)	83 (12.4)	31 (6.1)	113 (14.9)	61 (8.7)
Diagnosis of mental health disorders, n (%)							
Anxiety*	222 (5.1)	72 (8.2)	29 (3.6)	24 (3.6)	50 (9.8)	18 (2.4)	29 (4.1)
Mood disorders*	307 (7.1)	58 (6.6)	59 (7.3)	64 (9.6)	39 (7.6)	72 (9.5)	15 (2.1)
Personality disorders	3 (0.1)	0 (0)	0 (0)	1 (0.1)	0 (0)	2 (0.3)	0 (0)
Psychosis	88 (2.0)	27 (3.1)	14 (1.7)	7 (1.0)	14 (2.7)	18 (2.4)	8 (1.1)
Diagnosis of substance use disorder, n (%)							
Alcohol	72 (1.7)	20 (2.3)	19 (2.4)	6 (0.9)	2 (0.4)	19 (2.5)	6 (0.9)
Tobacco*	243 (5.6)	47 (5.4)	22 (2.7)	54 (8.1)	12 (2.3)	67 (8.8)	41 (5.9)
Opioid*	28 (0.6)	22 (2.5)	0 (0)	4 (0.6)	0 (0)	0 (0)	2 (0.3)
Cannabis*	39 (0.9)	0 (0)	10 (1.2)	14 (2.1)	1 (0.2)	3 (0.4)	11 (1.6)
Stimulants	41 (0.9)	8 (0.9)	13 (1.6)	3 (0.4)	2 (0.4)	14 (1.8)	1 (0.1)
Diagnosis of infectious disease, n (%)							
Hepatitis B*	178 (4.1)	25 (2.9)	0 (0)	8 (1.2)	24 (4.7)	27 (3.6)	94 (13.4)
Hepatitis C*	168 (3.9)	29 (3.3)	0 (0)	48 (7.2)	26 (5.1)	3 (0.4)	62 (8.9)
Chlamydia	15 (0.3)	0 (0)	12 (1.5)	1 (0.1)	2 (0.4)	0 (0)	0 (0)
Gonorrhea*	27 (0.6)	9 (1.0)	11 (1.4)	0 (0)	5 (1.0)	1 (0.1)	1 (0.1)
Syphilis*	413 (9.6)	67 (7.7)	117 (14.6)	27 (4.0)	53 (10.4)	92 (12.1)	57 (8.2)
Documented social history of sexual behaviors, n (%)							
Sexually Active*	725 (16.8)	180 (20.6)	0 (0)	0 (0)	0 (0)	545 (71.9)	0 (0)
Female partner*	236 (5.5)	163 (18.6)	0 (0)	0 (0)	0 (0)	73 (9.6)	0 (0)
Male partner*	741 (17.2)	4 (0.5)	0 (0)	0 (0)	0 (0)	737 (97.2)	0 (0)
Uses condom*	377 (8.7)	70 (8.0)	0 (0)	0 (0)	0 (0)	307 (40.5)	0 (0)
Documented social history of substance use, n (%)							
Alcohol*	677 (15.7)	325 (37.2)	0 (0)	0 (0)	0 (0)	352 (46.4)	0 (0)
Tobacco*	484 (11.2)	269 (30.8)	0 (0)	0 (0)	0 (0)	215 (28.4)	0 (0)
Illicit Drugs*	393 (9.1)	196 (22.4)	0 (0)	0 (0)	0 (0)	197 (26.0)	0 (0)
IV Drugs	4 (0.1)	4 (0.5)	0 (0)	0 (0)	0 (0)	0 (0)	0 (0)
Positive Laboratory result, n (%)							
Gonorrhea*	62 (1.4)	8 (0.9)	36 (4.5)	3 (0.4)	9 (1.8)	6 (0.8)	0 (0)
Chlamydia*	62 (1.4)	9 (1.0)	33 (4.1)	4 (0.6)	8 (1.6)	8 (1.1)	0 (0)
Syphilis*	496 (11.5)	81 (9.3)	197 (24.5)	23 (3.4)	89 (17.4)	63 (8.3)	43 (6.2)
Trichomoniasis*	32 (0.7)	6 (0.7)	1 (0.1)	12 (1.8)	0 (0)	13 (1.7)	0 (0)
Retention History, n (%)*							
Was retained in the previous year	3332 (77.2)	685 (78.4)	543 (67.5)	520 (77.6)	382 (74.8)	624 (82.3)	578 (82.7)
Was LTFU in the previous year	164 (3.8)	28 (3.2)	48 (6.0)	19 (2.8)	20 (3.9)	27 (3.6)	22 (3.1)
Previous visit was first visit	820 (19.0)	161 (18.4)	213 (26.5)	131 (19.6)	109 (21.3)	107 (14.1)	99 (14.2)

^*^P < 0.001

**Table 3:** Distribution of topics in the overall cohort as well across the six clinical phenotypes. Only topics that were observed to occur within clinical notes are shown below

Topics	Overall (n = 4316)	Class 1 (n = 874)	Class 2 (n = 804)	Class 3 (n = 670)	Class 4 (n = 511)	Class 5 (n = 758)	Class 6 (n = 699)
Opportunistic Infection, n (%)	1200 (27.8)	211 (24.1)	210 (26.1)	220 (32.8)	136 (26.6)	205 (27.0)	218 (31.2)
Comorbidities, n (%)*	2824 (65.4)	570 (65.2)	560 (69.7)	379 (56.6)	338 (66.1)	478 (63.1)	499 (71.4)
Poor Adherence, n (%)	32 (0.7)	10 (1.1)	7 (0.9)	7 (1.0)	1 (0.2)	4 (0.5)	3 (0.4)
Good Adherence, n (%)*	817 (18.9)	130 (14.9)	88 (10.9)	117 (17.5)	92 (18.0)	216 (28.5)	174 (24.9)
Sexual and gender minorities, n (%)*	1015 (23.5)	130 (14.9)	403 (50.1)	7 (1.0)	203 (39.7)	134 (17.7)	138 (19.7)
Heterosexual, n (%)*	683 (15.8)	188 (21.5)	97 (12.1)	168 (25.1)	59 (11.5)	98 (12.9)	73 (10.4)
Life stressors and markers of socioeconomic status,n (%)	1743 (40.4)	371 (42.4)	369 (45.9)	267 (39.9)	195 (38.2)	291 (38.4)	250 (35.8)
Mental illness, n (%)*	2135 (49.5)	445 (50.9)	414 (51.5)	357 (53.3)	273 (53.4)	391 (51.6)	255 (36.5)
Pregnancy, n (%)*	361 (8.4)	44 (5.0)	2 (0.2)	164 (24.5)	1 (0.2)	150 (19.8)	0 (0)
Preventive Health Services, n (%)*	3619 (83.9)	727 (83.2)	669 (83.2)	600 (89.6)	445 (87.1)	645 (85.1)	533 (76.3)
STI, n (%)*	1906 (44.2)	359 (41.1)	406 (50.5)	332 (49.6)	207 (40.5)	399 (52.6)	203 (29.0)
Substance use disorder,n (%)*	973 (22.5)	194 (22.2)	248 (30.8)	133 (19.9)	71 (13.9)	181 (23.9)	146 (20.9)

*P < 0.001

Class 2 patients, representing 19% of the total cohort, have a higher proportion of younger males, and have a lower proportion of comorbidities but have higher proportions of diagnosis codes for syphilis or positive laboratory results for syphilis. No one in this class was diagnosed with hepatitis B or C. Class 2 has the largest proportion of new patients, with 26% of patients in this class having no previous HIV care visits during the time period. Among clinical topics, approximately half of the patients in Class 2 had mentions of the “sexual/gender minorities” topic. In addition, patients in Class 2 had fewer mentions of the topic “good adherence.”

Class 3 patients, representing approximately 16% of the population, contain more females and a higher proportion of diagnosis codes indicating pregnancy. In addition, they have higher mentions of the topic “pregnant” and have lower mentions of the topic “sexual/gender minority.”

Class 4 patients, representing approximately 12% of the population, include more males and white and Hispanic patients. They also have a higher proportion of anxiety diagnoses (10% compared to 5% in the overall cohort). These patients had the lowest numbers of substance use disorder diagnoses, no documented social history of substance use, and had fewer mentions of the topic “substance use disorder.”

Patients belonging to Class 5, representing 18% of the overall cohort, had a more even split of male (42%) and female patients (58%) compared to the other classes. These patients had a higher proportion of documented social history of being sexually active, using condoms, and having male partners. Class 5 patients also had a higher documented history of substance use (similar to class 1) and more mentions of the clinical topic “pregnancy” (similar to class 3), but also had more mentions of the clinical topic “good adherence” compared to these and other classes.

Finally, patients belonging to Class 6 are older males who have diagnosis codes for chronic or infectious diseases such as cancer, diabetes, or hypertension and Hepatitis B or C compared to other classes. These patients, representing 16% of the overall cohort, have fewer mentions of the topic “mental illness” or “STI.”

### Outcome Analysis

The distributions of LTFU and high viral load are presented in Table 4. Class 2 had the greatest proportion of both LTFU and elevated HIV viral load among all classes (LTFU: 18.5%; elevated viral load: 39.8%). Class 4 had a high proportion of LTFU (16.8%) but had the lowest proportion of people with unsuppressed viral loads (19.8%). Table 4 also depicts the odds ratios for both outcomes across all classes, with Class 1 as the reference group. Compared to Class 1, patients in Class 2 had higher odds of being LTFU (OR 1.57, 95% CI [1.20–2.05], P = 0.001) and having an unsuppressed viral load (OR 1.74, 95% CI [1.42–2.13], P < 0.001). Patients in Class 4 had greater odds of being LTFU (OR 1.39, 95% CI [1.02–1.89], P = 0.034) but reduced odds of having an elevated viral load (OR 0.65, 95% CI [0.50–0.84], P = 0.001). Supplementary Table 3depicts the distribution of LTFU (based on the NHAS definition) and the corresponding odds ratios for the NHAS LTFU outcome across all classes, with Class 1 serving as the reference group. Compared to the primary LTFU definition, higher rates of the NHAS LTFU outcomes were observed across all classes (35.6%-48.4%). Similar to the results observed using the primary outcome, Class 2 had the highest prevalence of NHAS LTFU outcomes (48.4%) and the highest odds of experiencing the NHAS LTFU outcome (OR: 1.39, 95% CI [1.15–1.69], P < 0.001). Class 4 also appears to be at high risk for experiencing LTFU across both definitions (NHAS LTFU prevalence, 46.2%; OR, 1.27, 95% CI, [1.02–1.59], P = 0.032). Interestingly, when considering the NHAS definition, Class 3 patients had similar rates of LTFU as Class 4 patients (46.7%) and a similar odds ratio (1.30, 95% CI [1.06–1.59], P = 0.011), a trend which was not observed when using the primary LTFU definition (see Table 3). Supplementary Table 4 depicts the distribution of total visits and visits that were associated with LTFU (according to the primary definition and the NHAS definition) for each year in the study period. As can be seen, the number of visits decreased during the study period, with the COVID-19 pandemic year (2020) associated with a higher LTFU rate.

**Table 4:** Distribution of outcomes across the six clinical phenotypes

Class	Patient visits who were LTFU, n (% of class size)	OR (95% CI) for LTFU	Patient visits with high viral load, n (% of class size)	OR (95% CI) for high viral load
Class 1 (n = 874)	111 (12.7)	Ref.	241 (27.6)	Ref.
Class 2 (n = 804)	149 (18.5)*	1.57 (1.20–2.05)	320 (39.8)*	1.74 (1.42–2.13)*
Class 3 (n = 670)	92 (13.7)	1.09 (0.81–1.47)	186 (27.8)	1.01 (0.81–1.26)
Class 4 (n = 511)	86 (16.8)	1.39 (1.02–1.89)	101 (19.8)*	0.65 (0.50–0.84)
Class 5 (n = 758)	82 (10.8)	0.83 (0.61–1.13)	241 (31.8)	1.22 (0.99–1.52)
Class 6 (n = 699)	100 (14.3)	1.15 (0.86–1.53)	213 (30.5)	1.15 (0.92–1.43)

*P < 0.001

Also shown are the odds ratios for patients in each clinical phenotype experiencing each outcome compared to patients in Class 1

## Discussion

We identified a six-class model of clinically relevant phenotypes for patients with HIV, with good class separation and interpretability based on EHR and clinical notes. Our derived classes had the following distinctions: (1) documented social history of substance use, a high proportion of cardiopulmonary comorbidities; (2) younger males, newer patients, laboratory results for syphilis, diagnosis of syphilis; (3) female, pregnant; (4) non-Black, low substance use; (5) sexually active patients.; (6) older males with comorbidities. Clinical text topics supported these characteristics. We highlight that patients of Class 2 had the highest proportion of poor HIV care outcomes, including LTFU as well as elevated HIV viral load. Interestingly, patients in Class 4 had a high proportion of LTFU but a low proportion of patients with an elevated HIV viral load. Our phenotyping approach may inform health systems by giving a different view of their patient population that may impact their treatment.

PWH subgroup identification has been conducted using molecular profiling data, but few studies have detected clinical phenotypes among PWH using non-biological features. In a recent study, Mody et al. utilized LCA to group PWH with similar reasons for care disruptions, and detected five profiles of care disruptions that were strongly associated with current engagement status [27]. Follow-up studies from the same group also identified four subgroups of PWH that differed by client-provider communication profiles using LCA [28]. In another study, Fernandez et al. utilized LCA to detect three groups of PWH based on housing status [29]. One of these groups, women who predominantly lacked stable housing and did not disclose HIV status, had a higher incidence of poor outcomes, such as not being retained in care or having an unsuppressed viral load. Latent profile analysis has also been used to detect profiles of socio-cognitive facilitators of ART adherence [30]. Our study adds to this body of work by identifying six latent classes using structured and unstructured data within the EHR from PWH clinic visits to examine LTFU.

Individuals in Class 1 were more likely to use substances, including alcohol and tobacco, as well as to have cardiovascular or pulmonary disease. The association between substance use and cardiopulmonary disease is not surprising, as studies have clearly shown that tobacco and alcohol use increase the risk for cardiovascular disease [31]. Tobacco use has also been strongly linked to lung diseases such as chronic obstructive pulmonary disease and lung cancer [32, 33]. PWH have higher rates of smoking than the general population and are at higher risk for tobacco-associated health outcomes such as cardiovascular disease and lung cancer than people who smoke but do not have HIV [34–36]. Elevated HIV viral load can cause systemic inflammation and exacerbate cardiovascular risk [36], so it is reassuring that Class 1 patients did not have higher rates of elevated HIV viral loads compared to other classes.

Individuals in Class 2 were more likely to be young males, new patients to the clinic, and diagnosed with syphilis. These individuals were also more likely to be LTFU and have an elevated HIV viral load. Syphilis diagnoses have dramatically increased in the United States in the past decade, particularly among MSM [37]. Other studies have also found that younger people are more likely to lapse in HIV care and have elevated viral load than those who are older [11, 38–41]. Those who were new to the clinic may have also presented with an elevated viral load over the next year because they had previously been out of care or were newly diagnosed with HIV and not yet on ART. Those in Class 2 may benefit from evidence-based intervention to improve retention in care among young men with HIV [42, 43].

Class 3 patients were more likely to be pregnant women, who were more likely to be LTFU according to the NHAS definition, compared to the primary LTFU definition. This may be due to the fact that women with HIV often receive both HIV care and prenatal care during their pregnancy, but after delivery, mothers are more likely to be LTFU [44–46]. A single appointment within 12 months is more likely to identify pregnant women who exhibit this pattern of retention during pregnancy and LTFU after delivery than the NHAS definition, which requires two visits separated by 90 days, for the definition of retention [47, 48].

Class 4 individuals had relatively high rates of LTFU but low rates of elevated HIV viral loads. This finding may seem somewhat counterintuitive, as the literature has shown that viral non-suppression and LTFU are often correlated [24], as was the case with individuals in Class 2. However, these Class 4 individuals may represent a group that is not scheduled for clinical visits as frequently as other groups but are still in contact with their providers and receiving refills for ART, resulting in well-controlled HIV and viral suppression [49]. Individuals in Class 4 were more likely to be white or Hispanic, to have no documented history of substance use, or to have anxiety. Studies have shown that substance use disorder (SUD) disproportionately affects minoritized groups of PWH, with non-Hispanic Black PWH having a higher likelihood of SUD compared to non-Hispanic White PWH [50]. Our finding that Class 4 individuals had low rates of substance use and high viral suppression is consistent with other studies that have also shown that SUD is associated with a higher risk for elevated HIV viral load among PWH [51]. Furthermore, these individuals were also more likely to have a diagnosis of anxiety. Anxiety is common among PWH, and screening for and treating anxiety in PWH can improve clinical outcomes. PWH with treated anxiety have similar HIV care outcomes to those without anxiety, but those with untreated moderate to severe anxiety are more likely to have viral non-suppression [52]. It is possible that those in Class 4 have treated anxiety, as their providers diagnosed it.

As our clinical phenotype groups are distinct by characteristics, the results from our study could provide the ability to design personalized interventions to prevent LTFU or increase re-linkage to care. Groups may have different reasons for LTFU and/or specific barriers to re-linkage that can be addressed differently. For example, patients in Class 2 who were younger but had the highest risk of both outcomes may benefit from interventions that align with current technological trends, e.g., reminders of appointments through phone-based apps or instant messaging instead of direct phone calls. In contrast, older patients in Class 6 may benefit from more human interactions when targeted to prevent LTFU. Furthermore, our results suggest that to improve outcomes, HIV care engagement may be initiated during routine check-ups or disease- or condition-related appointments for patients in Class 1 (cardiopulmonary conditions), Class 3 (pregnant women), or Class 6 (cancer or liver disease). Finally, our description of clinical phenotypes could inform future studies on PWH. For example, studies focused on developing prediction models could use our phenotype descriptors to test model performance within specific patient subgroups of PWH as an additional sensitivity analysis. Our phenotype descriptions could also inform patient recruitment efforts for future randomized controlled trials to achieve a more diverse and clinically nuanced population.

Our study has limitations. Due to the single-center, retrospective nature of our study, the derived clinical phenotypes may not be generalizable to future patients or those in other clinics. Our cohort comprises a mix of first-time visits, which may include newly diagnosed or transferred patients, as well as active patients in our clinic. However, we have adjusted for retention history, which indicates if a patient was retained in the previous year or if the previous visit was the first. By not separating these categories, our results demonstrate the real-world applicability, as any prospective visit can be identified as one of six PWH phenotypes, with corresponding clinical relevance and disease management implications. However, prospective and external validation studies remain an area of future work. An additional limitation is that the LCA algorithm resulted in each patient visit being assigned to a single latent class. While our approach was guided by the objective of interpreting the detected latent classes, it is possible that a patient may be represented by more than one class. Advanced probabilistic models, such as mixture models, that allow the representation of a patient as a combination of different classes, may yield interesting results and remain an area of focus for future work. Another limitation is that our results may be confounded by unadjusted biases from covering pre-pandemic (3 years) and COVID-19 pandemic (1 year) phases. We observed a higher rate of LTFU during the pandemic period, which is in line with published studies [53]. However, our clinic offered telehealth options in 2020, and telehealth visits were counted in our LTFU outcome. Notably, our primary definition of being LTFU is based on a single follow-up visit and is therefore less sensitive to changes related to the COVID-19 pandemic. Finally, it is also possible that some of our LTFU patients may have continued to seek care elsewhere. However, this is likely to be a small population, as in recent work, there was found to be a 4% rate of patients with HIV transferring care among a cohort built from multiple hospitals in the Chicagoland area [39].

## Conclusion

Using a latent class analysis of patient visits at an urban HIV care clinic, we detected six distinct and clinically relevant phenotypes of PWH: cardiopulmonary chronic patients with a history of substance use (Class 1), younger males or newer patients with sexually transmitted infection (Class 2), pregnant women (Class 3), non-Black patients with low substance use (Class 4), sexually active (Class 5), and older males with chronic conditions (Class 6). The detection of these phenotypes should inform interventional strategies and future research to improve care and outcomes among PWH.

## Supplementary Information

Below is the link to the electronic supplementary material.

Supplementary file1 (DOCX 23 KB)

## Data Availability

Data and code are available upon reasonable request to the corresponding author.
