# Supplementary material for: Identification of Clinical Phenotypes Among People with HIV Using Electronic Health Record Data
Source: AIDS Behav. 2025 Oct 6;30(2):454–63. doi: 10.1007/s10461-025-04893-7 (PMC12929249; doi:10.1007/s10461-025-04893-7)
Supplement: Supplementary file 1 — Supplementary file1 (DOCX 23 KB) [file 10461_2025_4893_MOESM1_ESM.docx]

**Supplementary Table 1**: List of EHR variables used for phenotyping

| Variable | Details |
| --- | --- |
| Demographics | Age (binned into ≤25, 26-35, 36-45, 46-55, and >55 years), race, ethnicity, gender |
| Diagnosis Codes | Codes related to   - Comorbidities (Cancer, Cardiovascular Diseases, Diabetes, Pulmonary conditions, Hypertension, Kidney diseases, Liver disease) - Mental Health (Anxiety, Mood Disorders, Personality Disorders, Psychosis) - Pregnancy - Obesity - Substance Use (Alcohol, Tobacco, Opioid, Cannabis, Stimulants), - Infectious diseases (Hepatitis B, C) - Sexually Transmitted Diseases (Chlamydia, Gonorrhea, Syphilis, or Sexually Transmitted Infection) |
| Social History | Sexual history (active, gender of partner, condom use) , substance use history (alcohol, tobacco, illicit drugs, injection drugs) |
| Laboratory Results | STI lab results (gonorrhea, chlamydia, syphilis, trichomoniasis) |
| Retention history | Retained in the previous year, previous visit was first visit. |

**Supplementary Table 2**: List of clinical terms used for phenotyping. Text within clinical notes was searched for any mentions of tokens. Each topic was indicated as being present for a clinic appointment if any associated tokens were found if it was not accompanied by an exclusion token +/- 3 words before or after the token mention. Table is taken from Oliwa T, Furner B, Schmitt J, Schneider J, and Ridgway JP. “Development of a predictive model for retention in HIV care using natural language processing of clinical notes.” J Am Med Inform Assoc. 2021 Jan 15;28(1):104-112, by permission of Oxford University Press.

| **Clinical topics** | **Associated word tokens** |
| --- | --- |
| HIV genotype mutation | “k103,” “k103n,” “m184v,” “resistant hiv,” “resistant virus” |
| Congenital HIV | “congenital hiv,” “congenital infection,” “perinatal,” “since birth,” “vertical transmission” |
| Injection drug use | “heroin,” “idu,” “intravenous drug use,” “ivdu” |
| Opportunistic infection | “burkitt,” “candidiasi,” “candidiasis,” “cmv,” “cmv retinitis,” “cryptococcal,” “cryptococcus,” “cryptosporidium,” “cytomegalovirus,” “jc virus,” “kaposi,” “ks,” “lymphoma,” “mac,” “mai,” “mycobacterium avium,” “pcp pneumonia,” “pjp pneumonia,” “pml,” “pneumocystis,” “thrush,” “toxo,” “toxoplasmosis” |
| Comorbidities | “hbv,” “hcv,” “hepatitis b,” “hepatitis c,” “tb,” “tuberculosis” |
| Poor adherence | “bad adherence,” “difficulty with adherence,” “do not fill,” “frequent miss dose,” “medication access,” “miss appointment,” “miss appt,” “no show,” “non adherence,” “non adherent,” “non compliant,” “nonadherence,” “nonadherent,” “noncompliant,” “not adherent,” “off art,” “off haart,” “out of med,” “poor adherence,” “poor compliance,” “run out,” “sometimes forget,” “unable to fill,” “without med” |
| Condomless sex | “condom use no,” “no condom,” “not use condom,” “unprotected,” “unprotected sex,” “without condom” |
| Good adherence | “adherent,” “compliant,” “do well on,” “excellent adherence,” “good adherence,” “never miss,” “no miss dose,” “well on art” |
| Sex with condoms | “condom use yes,” “use condom” |
|  |  |
| Sexual and gender minorities | “lgbt,” “lgbtq,” “man who have,” “msm,” “transwoman” |
| Heterosexual | “hetero,” “heterosexual” |
| Life stressors and markers of socioeconomic status | “adap,” “afc,” “case management,” “case manager,” “court date,” “death in family,” “disability,” “disclosure,” “ed visit,” “emergency room,” “financial support,” “fmla,” “homeless,” “homophobia,” “house arrest,” “incarcerate,” “incarceration,” “insurance issue,” “insurance lapse,” “jail,” “kick out,” “lawyer,” “life alone,” “live alone,” “medicaid lapse,” “new phone number,” “no family,” “not disclose,” “not discuss status,” “partner unaware,” “phone break,” “prison,” “recent death,” “recent ed visit,” “redetermination,” “rent assistance,” “stigma,” “stress,” “stressor,” “sw call pt,” “transportation,” “unemployed,” “uninsured,” “unstable housing,” “ventra,” “vital bridge,” “wic,” “without insurance coverage” |
| Mental illness | “anxiety,” “anxious,” “behavioral health,” “bh referral,” “bipolar,” “c2p,” “care prevent,” “care2prevent,” “cry,” “deny si,” “depress,” “depressed,” “depression,” “emotional,” “grief,” “insomnia,” “panic,” “passive si,” “psychiatrist,” “psychiatry,” “psychosis,” “psychotic,” “sad,” “schizophrenia,” “sleepless,” “suicidal,” “suicide,” “tearful,” “therapist” |
| Pregnancy | “c section,” “cesarean section,” “pregnancy,” “pregnant,” “vaginal delivery” |
| Preventive health services | “administer pneumococcal,” “colonoscopy,” “flu shot,” “human papillomavirus vaccine,” “influenza vaccine,” “mammogram,” “menactra,” “pap,” “pap smear,” “pcv13,” “pneumovax,” “prevnar,” “psv23,” “quadrivalent,” “tdap,” “trivalent,” “vaccine” |
| Married/partnered | “husband,” “married,” “marry,” “monogamous,” “wife” |
| Social support | “church,” “prayer,” “support group” |
| STI | “benzathine,” “bicillin,” “chancroid,” “chlamydia,” “chlamydia trachomatis,” “crab,” “crab louse,” “gc,” “genital herpe,” “gonorrhea,” “granuloma inguinale,” “haemophilus ducreyi,” “herpes,” “herpes simplex,” “hpv,” “hsv,” “hsv1,” “hsv2,” “human papillomavirus,” “klebsiella granulomatis,” “lgv,” “lymphogranuloma venereum,” “neisseria gonorrhoeae,” “pediculosis pubis,” “pelvic inflammatory disease,” “penicillin g,” “pid,” “positive rpr,” “pthirus pubis,” “pubic lice,” “std,” “sti,” “syphilis,” “treponema pallidum,” “trich,” “trichomona vaginalis,” “trichomoniasis,” “valacyclovir,” “valtrex” |
| Substance use disorder | “aa meeting,” “alcohol abuse,” “alcoholic,” “amphetamine abuse,” “beer,” “cocaine,” “crack,” “crystal meth,” “drug treatment program,” “drunk,” “haymarket,” “heroin,” “intravenous drug user,” “ivdu,” “ivu,” “marijuana,” “meth,” “methadone,” “methamphetamine abuse,” “na meeting,” “narcotic,” “sober,” “substance abuse,” “substance use,” “take drug” |
| Exclusion | “no,” “not,” “none,” “neg,” “never,” “negative,” “non,” “deny,” “denies,” “father,” “dad,” “mother,” “mom,” “brother,” “brothers,” “sister,” “sisters,” “sibling,” “siblings,” “cousin,” “cousins,” “aunt,” “aunts,” “uncle,” “uncles,” “grandmother,” “grandparent,” “grandparents,” “grandfather,” “grandchild,” “grandchildren,” “grandson,” “grandsons,” “granddaughter,” “granddaughters,” “wife,” “spouse,” “husband,” “child,” “children,” “offspring,” “progeny,” “son,” “sons,” “daughter,” “daughters,” “nephew,” “nephews,” “niece,” “nieces,” “kin” |

**Supplementary Table 3**: Distribution of the LTFU outcome, according to the NHAS definition, across the six clinical phenotypes. Also shown are the odds ratios for patients in each clinical phenotype experiencing the NHAS LTFU outcome compared to patients in Class 1.

| Class | Patient visits who were LTFU by the NHAS definition, n (% of class size) | OR (95% CI) for HRSA definition |
| --- | --- | --- |
| Class 1  (n=874) | 352 (40.3) | Ref |
| Class 2  (n=804) | 389 (48.4)* | 1.39 (1.15-1.69)* |
| Class 3  (n=670) | 313 (46.7) | 1.30 (1.06-1.59) |
| Class 4  (n=511) | 236 (46.2) | 1.27 (1.02-1.59) |
| Class 5  (n=758) | 270 (35.6) | 0.82 (0.67-1.00) |
| Class 6  (n=699) | 289 (41.3) | 1.04 (0.85-1.28) |

**Supplementary Table 4**: Distribution of total number of visits and visits associated with LTFU for each year in the study period. Both definitions of LTFU (primary: >365 days elapsed between successive HIV clinic visits; NHAS: no successive HIV care visits that were 90 days apart within a 365-day period).

| Year | # HIV clinic visits | # HIV clinic visits associated with LTFU (primary definition) | # HIV clinic visits associated with LTFU (NHAS definition) |
| --- | --- | --- | --- |
| 2017 | 1,196 | 172 (14.4%) | 486 (40.6%) |
| 2018 | 1,109 | 121 (10.9%) | 401 (36.2%) |
| 2019 | 1,103 | 171 (15.5%) | 502 (45.5%) |
| 2020 | 908 | 156 (17.2%) | 460 (50.7%) |
| All years | 4,316 | 620 (14.4%) | 1,849 (42.8%) |
